# Insulin-stimulated glucose uptake is impaired in senescent human adipocytes

**DOI:** 10.3389/fendo.2026.1795654

**Published:** 2026-03-17

**Authors:** Ida Alexandersson, Henrik Palmgren, Martin Uhrbom, Jan Oscarsson, Jeremie Boucher

**Affiliations:** 1 Bioscience Metabolism, Research and Early Development, Cardiovascular, Renal and Metabolism (CVRM), BioPharmaceuticals R&D, AstraZeneca, Gothenburg, Sweden; 2 Department of Molecular and Clinical Medicine, Institute of Medicine, Sahlgrenska Academy, University of Gothenburg, Gothenburg, Sweden; 3 Bioscience Renal, Research and Early Development, Cardiovascular, Renal and Metabolism, BioPharmaceuticals R&D, AstraZeneca, Gothenburg, Sweden; 4 Assays, Profiling & Cell Sciences (APCS), Discovery Sciences, AstraZeneca, Gothenburg, Sweden; 5 Biopharmaceuticals R&D, Late−Stage Clinical Development, Cardiovascular, Renal and Metabolism, AstraZeneca, Gothenburg, Sweden; 6 Metabolic Diseases, Evotec International GmbH, Göttingen, Germany

**Keywords:** adipocytes, cellular senescence, glucose uptake, insulin sensitivity, lipolysis

## Abstract

**Background:**

Cell senescence, a state of cell cycle arrest induced by intrinsic or extrinsic stress, is linked to aging and aging-associated diseases. Senescence markers are elevated in adipose tissue with age and in obesity. Recently, it was shown that human mature adipocytes can undergo senescence in response to hyperinsulinemia. However, the functional consequences of adipocyte cell senescence remain poorly understood.

**Methods:**

To study the impact of cellular senescence on human adipocyte function, we induced senescence in differentiated primary human preadipocytes (referred to as human adipocytes) using nutlin-3a, doxorubicin and etoposide. Expression of senescence markers, insulin receptor signaling, response to lipolytic stimuli and insulin mediated glucose uptake were investigated.

**Results:**

The senescence-inducing compounds increased expression of senescence markers p21, p53, activity of senescence-associated β-galactosidase (SA-βgal) and secretion of senescence-associated secreted factors (SASP). We showed that insulin-stimulated glucose uptake, but not basal glucose uptake, was significantly reduced in senescent adipocytes. Insulin receptor signaling was largely unaffected, while expression of insulin receptor signaling proteins and especially GLUT4 expression were reduced. Expression of some adipocyte marker genes, including *ADIPOQ, LEP*, *PNPLA2, LIPE* and *PPARγ2*, and secretion of adiponectin were reduced in the senescent adipocytes. In contrast, lipolytic capacity of senescent adipocytes was largely unaffected.

**Conclusions:**

Our findings indicate that cellular senescence impairs insulin-stimulated glucose uptake but not lipolytic capacity; changes that may contribute to impaired glucose control and ectopic fat accumulation.

## 1 Introduction

Cellular senescence has emerged as a contributor to age-related diseases, including type 2 diabetes (T2D) (1, 2). It is a cellular state of proliferative arrest induced by various stressful stimuli including DNA damage, oncogene activation, telomere shortening, and reactive oxygen species (3). The senescent phenotype is complex, with senescent cells commonly displaying altered morphology, elevated activity of senescence-associated β-galactosidase (SA-βgal), an active DNA damage response, resistance to apoptosis, increased expression of cyclin-dependent kinase inhibitors p21^CIP1^ and p16^INK4a^, and tumor suppressor p53, as well as a senescence-associated secretory phenotype (SASP) (4, 5). The pro-inflammatory SASP involves secretion of various factors and molecules including cytokines, chemokines, growth factors and proteases. SASP can modulate multiple processes including trigger inflammatory cascades and affect the function of neighboring non-senescent cells (6, 7). Transplantation studies in mice have demonstrated that less than 0.03% senescent cells in the body is sufficient to induce physical dysfunction, whereas clearance of senescent cells using transgenic mouse models or senolytics improve physical and metabolic health (8–10).

White adipose tissue (WAT) is a critical organ in the regulation of whole-body metabolic health both as a regulator of glucose and lipid homeostasis, and as an endocrine organ (11). Dysfunctional WAT is central in the development of insulin resistance – a common characteristic in both obesity and T2D. WAT acts as a storage site of triglycerides, but the capacity of WAT to expand in response to excess energy intake is limited. Expansion of WAT beyond its capacity results in increased adipocyte cell size and ectopic lipid accumulation in other organs, chronic inflammation, and insulin resistance (12).

In adipose tissue, cell senescence increases with age and in obesity – both of which are risk factors for the development of T2D (13–17). Furthermore, cellular senescence has been linked to the impairment of proper WAT expansion. Markers of senescence are increased in subcutaneous adipose tissue (SAT) in individuals with hypertrophic obesity (15), and co-cultures have demonstrated that senescent preadipocytes (adipocyte progenitor cells) impede adipogenesis of non-senescent neighboring preadipocytes (6). It was recently shown that human mature adipocytes, which are considered postmitotic cells, can re-enter the cell cycle and senesce in response to hyperinsulinemia (18). Since then, it has been demonstrated that markers of senescence are increased in mature adipocytes isolated from obese individuals and further increased in individuals with obesity and T2D (17). However, our understanding of the consequences of cellular senescence on human adipocyte phenotype and function is limited.

Here, we explore the impact of cellular senescence on human adipocyte metabolic function and possible links to metabolic disease. Our findings show that senescent adipocytes become dysfunctional with reduced insulin-stimulated glucose uptake and reduced expression of adipocyte differentiation markers, whereas lipolytic activity and insulin signaling remain largely intact.

## 2 Materials and methods

### 2.1 Human adipose samples

Anonymous samples of SAT were obtained as a surgical biopsy from the abdominal region of non-obese female individuals undergoing elective surgery at Sahlgrenska University Hospital in Gothenburg, Sweden. All study subjects received written and oral information before giving written informed consent for the use of their tissue. The study was approved by the Regional Ethical Review Board in Gothenburg, Sweden. A description of the patient cohort used in the study can be found in **Supplementary Table 1**.

### 2.2 Isolation of preadipocytes from human adipose tissue

Human SAT was processed for adipose cell isolation as described previously (19, 20). After separating the infranatant containing the stromal vascular fraction (SVF) from the floating adipocytes, the SVF was pelleted by centrifugation (200x*g*, 7 min). To remove red blood cells, the pellet was incubated (RT, 5 min) in red blood cell lysing buffer (Sigma-Aldrich, Cat. R7757) followed by centrifugation (200x*g*, 5 min). The pellet was resuspended in subcutaneous basal medium (BM-1; ZenBio, BM-1), 10% FBS (Gibco, Cat. 10270-106), 1% penicillin and streptomycin (Pen/Strep), and 1 nM bFGF (Sigma-Aldrich, Cat. F0291). The cells were seeded at a density of 20,000 cells/cm^2^ and expanded in flasks or cell factories. The cells were cultured at 37 °C with 5% CO_2_ and 95% relative humidity.

### 2.3 Differentiated human adipocyte cultures and compound treatment

Human preadipocytes from 10 female adults were isolated from human SAT surgical biopsies (n = 7) or purchased from Lonza (n = 3) as Human Adipose-derived stem cells from liposuction (Lonza, Cat. PT-5006). The preadipocytes were seeded for experimental use in subcutaneous preadipocyte medium (PM-1; ZenBio, PM-1) supplemented with 1 nM bFGF. Medium was changed the following day to PM-1 supplemented with 1 nM bFGF and 15 ng/ml BMP4 (R&D systems, Cat. 314-BP-010/CF). After becoming confluent (usually after 4–5 days), differentiation was initiated by changing the medium to subcutaneous basal medium (BM-1; ZenBio, BM-1), 3% FBS (Gibco, Cat. 10270-106), 1% Pen/Strep, 1 µM dexamethasone (Sigma-Aldrich, Cat. D2915), 0.5 mM IBMX (Sigma-Aldrich, Cat. I5879), 100 nM insulin (Actrapid Penfill; Novo Nordisk), and 1 µM pioglitazone (AstraZeneca compound management). Medium was changed on day 4. On day 7 of differentiation, the medium was changed but with the exclusion of IBMX and pioglitazone. On day ten of differentiation, a medium change was done and 5 µM nutlin-3a (Sigma-Aldrich, Cat. SML0580), 50 µM etoposide (Sigma-Aldrich, Cat. E1383) or 0.3 µM doxorubicin (AstraZeneca compound management) was added to the cells to induce senescence. The specified concentrations were chosen from pilot studies set up to define the concentrations to induce senescence without affecting cell viability in the human adipocytes (**Supplementary Figures 1A, B**). Cytotoxicity was assessed by alamarBlue staining. Medium was changed again 4 days later. Further analyses were performed after 7 days of treatment, unless indicated differently. As a control, 0.1% dimethylsulfoxide (DMSO) was used.

For washout experiments, on day 7 of treatment with nutlin-3a, etoposide and doxorubicin, adipocytes were washed twice with PBS. The cells were thereafter cultured for 4 days in BM-1 supplemented with 3% FBS, 1% Pen/Strep, 1 µM dexamethasone, 100 nM insulin, until further processing.

For the acute insulin signaling experiments, adipocytes were washed twice with PBS and starved of insulin and serum for 4 hours in DMEM (Gibco, Cat. 31966021), 25 mM HEPES (Gibco, Cat. 15630056), and 0.1% BSA (Sigma-Aldrich, Cat. A6003). The cells were incubated with 1 or 10 nM of insulin for 10 mins at 37°C with 5% CO_2_. Plates with adipocytes were put on ice, medium was removed followed by washing with cold PBS, and the cells were lysed in RIPA buffer for Western blot analysis.

### 2.4 Real-time PCR

Total RNA was isolated using the RNeasy Mini kits (Qiagen, Cat. 74106), following the manufacturer’s instructions. Complementary DNA (cDNA) was reverse transcribed, from RNA using the High Capacity cDNA Reverse Transcription Kit (Applied Biosystems, Cat. 4368814) and a heat cycler. Gene expression was measured using real-time PCR with SYBR Green PCR Master Mix (Applied Biosystems, Cat. 4309155) or TaqMan Fast Advanced Master mix (Applied Biosystems, Cat. 4444557) on a Quantstudio 7 Flex Real-Time PCR machine (Applied Biosystems). The following Real Time PCR protocols were used: For SYBR: polymerase activation (95°C, 10 min), followed by 40 cycles of denaturation (95°C, 15 s) and annealing/extension (60°C, 1 min), and TaqMan: polymerase activation (95°C, 20 s), followed by 40 cycles of denaturation (95°C, 1 s) and annealing/extension (60°C, 20 s). Tata-binding protein (TBP) was used as an internal normalization control. Primer sequences and probes are listed in **Supplementary Table 2**.

### 2.5 Western blot

To obtain protein extracts from differentiated adipocytes, cells were washed with PBS and lysed in RIPA buffer (20 mM Tris-HCl (pH 7.5), 150 mM NaCl, 1 mM EGTA, 1 mM EDTA, 1% Triton X-100, 0.1% SDS) containing protease inhibitors (Roche, Cat. 11836170001). For measurement of insulin signaling proteins, protease inhibitors, and PhosStop (Roche, Cat. 4906845001) were added to the lysis buffer. Lysates were snap-frozen and thawed rapidly at 90°C twice, and centrifuged (10,000x*g*, 5 min, 4°C). The clarified lysate was removed from lipids and pellet and was centrifuged (10,000x*g*, 5 min, 4°C) to remove all remaining carryover lipid. Protein concentration was measured using a BCA Protein Assay Kit (ThermoFisher Scientific, Cat. 23227). Proteins were separated on 4-12% Bis-Tris NuPAGE gels (Invitrogen) and transferred to PVDF membranes (Invitrogen, Cat. LC2005). Membranes were blocked for 60 min at RT in 1X TBST with 5% w/v nonfat dry milk and incubated overnight at 4°C with primary antibodies. Membranes were washed and incubated with secondary antibodies for 1 hour at RT. Bands were detected using ECL Western Blotting Substrate (Pierce, Cat. 32106) or SuperSignal West Femto (ThermoFisher Scientific, Cat. 34096) in a ChemiDoc MP Imaging system (Bio-Rad). Image Lab version 6.0.1 software (Bio-Rad) was used to quantify the bands. Quantified protein levels were normalized to β-actin, and phosphorylated protein levels are normalized to β-actin or respective total protein. Primary antibodies are listed in **Supplementary Table 3**.

### 2.6 Senescence-associated β-galactosidase activity

Assessment of SA-βgal activity was performed using a cellular senescence activity kit (Enzo Life Sciences, Cat. ENZ-KIT129-0120) according to the manufacturer’s instructions. In brief, cell lysates were incubated with SA-βgal substrate for 2 hours at 37°C. Fluorescence was read at 360 nm (excitation)/465 nm (emission) with a CLARIOstar (BMG LABTECH) plate reader. Protein concentration was measured in the cell lysates using a BCA Protein Assay Kit. Recorded relative fluorescence units were normalized to protein concentration.

### 2.7 Cell culture media protein analysis

Cell culture media was collected on day 7 of treatment with nutlin-3a, doxorubicin, and etoposide, and was filtered through a 0.2 µm syringe filter. The Olink^®^ Target 96 inflammation panel (Olink Proteomics AB), which enables simultaneous analysis of 92 analytes in 1 µL of sample, was used following manufacturer’s instructions. Out of 92 analytes, 39 were detected in at least 75% of the samples. Using statistical analyses, 32 analytes were found to display a significant difference between the groups, *P* < 0.05 (Friedman test), and were selected for further analysis. For the hierarchical clustering of 24 samples (six donors; four treatment groups), the R-package pheatmap (version:1.0.12) was used. Averaged, centered, and scaled values were used as input, rows (proteins) were ordered based on euclidean distances using the clustering method “complete”. For a detailed list, see **Supplementary Table 4**. Adiponectin levels were determined using an ELISA kit (R&D systems, Cat. DRP300) according to the manufacturer’s instructions.

### 2.8 Lipolysis

Lipolysis activity was measured by quantifying glycerol in cell culture medium after stimulation with forskolin or isoproterenol. Human preadipocytes were seeded, differentiated, and treated with nutlin-3a, doxorubicin and etoposide in 96-well plates (Corning, Cat. 3595). On day 7 of treatment with nutlin-3a, doxorubicin, and etoposide, human adipocytes were washed and incubated (37°C, 5% CO_2_, 3 hours) in sterile-filtered serum- and insulin-free medium (DMEM [Gibco, Cat. 31966], 1% BSA [Sigma-Aldrich, Cat. A8806], 1% Pen/Strep). Medium was changed to KRHB buffer (120 mM NaCl, 4.7 mM KCl, 1.2 mM KH_2_PO_4_, 1.2 mM MgSO_4_·7H2O, and 2.5 mM CaCl_2_·2H_2_O, 25 mM HEPES, 2 mM glucose, 1% BSA [Sigma-Aldrich, Cat. A6003], pH 7.4). Lipolysis was stimulated by adding forskolin (Sigma-Aldrich, Cat. F6886, 5 concentrations, 1:10 dilutions, final conc. 0.01-100 µM) or isoproterenol (Sigma-Aldrich, Cat. 16504-100MG, 5 concentrations, 1:10 dilutions, final conc. 0.01–100 nM) using a Biomek FX autosampler workstation (Beckman Coulter). The adipocytes were incubated for 2 hours (37°C, 5% CO_2_). The Biomek FX autosampler workstation was used to transfer 14 µL of medium in four replicates to a 384-well plate (Greiner Bio-one, Cat. 781101) containing a 6.25-200 µM glycerol standard curve (Sigma-Aldrich, Cat. G7793). Sixty microliters of Free Glycerol Reagent (Sigma-Aldrich, Cat. F6428) was added to each well followed by incubation (10 min, RT). The absorbance was analyzed at 540 nm using a Paradigm™ Detection Platform (Beckman Coulter). Quantified glycerol was normalized by total cell number (Hoechst staining).

### 2.9 Glucose uptake

Human preadipocytes were differentiated and treated with nutlin-3a, doxorubicin, and etoposide in CytoStar-T 96-well plates (Perkin Elmer, Cat. RPNQ0162). The adipocytes were washed twice and incubated (37°C, 5% CO_2_, 3 hours) in serum- and insulin-free media (70% DMEM [Gibco, Cat. 31966], 27% DMEM [Gibco, Cat. 11966], 25 mM HEPES [Gibco, Cat. Cat. 15630056], 0.1% BSA [Sigma-Aldrich, Cat. A6003]). Medium was changed to assay medium (DMEM [Gibco, Cat. 11966], 25 mM HEPES [Gibco, Cat. 15630056], 0.1% BSA [Sigma-Aldrich, Cat. A6003], 2 nM Sodium pyruvate [Gibco, Cat. 11360070]). Glucose uptake was stimulated by adding insulin (Novo Nordisk, 9 concentrations in 1:4 dilutions, final conc. 0.015 nM-1 µM) in 8 replicates using a Biomek FX autosampler workstation (Beckman Coulter) followed by incubation (37°C, 5% CO_2_, 45 min). Non-specific binding controls received cytochalasin B (Sigma-Aldrich, Cat. C2743, final conc. 20 µM). Twenty-five microliters of 2.5 μCi/ml 2-[1-^14^C]-Deoxy-D-Glucose (Perkin Elmer, Cat. NEC495A250UC) and the cells were incubated (37°C, 5% CO_2_, 10 min). Glucose uptake was stopped by addition of cytochalasin B (final conc. 20 µM), and the plate was read using a Microbeta2 plate reader (Perkin Elmer). Glucose uptake was normalized by total cell number (Hoechst staining). Cytochalasin B control values were subtracted.

### 2.10 Immunocytochemistry

For fluorescent analysis of ɣH2AX, p21 and lipid accumulation, human adipocytes were cultured and treated as previously described in 96-well plates (PerkinElmer, Cat. 6055302). Adipocytes were washed twice with PBS and fixed with 4% paraformaldehyde for 20 min at RT followed by washing thrice with PBS. For analysis of ɣH2AX and p21, cells were permeabilized using 0.1% Triton X-100 in PBS for 10 min at RT. After washing thrice with PBS, adipocytes were blocked in 2% BSA in PBS for 30 min at RT. The cells were incubated with primary antibody in 1% BSA/PBS for 60 min. After washing thrice with PBS, adipocytes were incubated with secondary antibody in 1% BSA/PBS for 60 min at RT and washed thrice with PBS. Adipocytes were counterstained with Hoechst (1:5000 in 1% BSA/PBS, ThermoFisher Scientific, Cat. 33342) for 30 min at RT followed by washing thrice with PBS. For fluorescent analysis of lipid accumulation, fixated cells were left to stain for 20 min in PBS containing 0.1 µg/ml BODIPY 493/503 (Invitrogen, Cat. D3922) and Hoechst (1:5000) followed by washing thrice with PBS. Images were acquired with a robotic Yokogawa CV8000 (ɣH2AX and BODIPY) or Yokogawa CV7000 spinning disc confocal microscope (Wako Automation) at 20× magnification (NA.75, 2 × 2 binning) using ZYLA 5.5 sCMOS cameras Andor Technology). Images were acquired with 16 bits image depth and a resolution of 1000 × 1000 (ɣH2AX and BODIPY) or 1280 x 1080 (p21), with a pixel dwell of ~1.02 μs. ɣH2AX, p21, and BODIPY intensity was quantified using Columbus 2.9.1.532 software (PerkinElmer). Primary and secondary antibodies are listed in **Supplementary Table 3**.

### 2.11 Statistical analysis

Results are presented as mean ± SEM. The number of replicates, shown as dots, represents individual donors and are stated in figure legends of each measurement. For comparisons between three or more groups, a Friedman test for non-parametric analysis with the assumption of non-normally distributed data was performed, followed by Dunn’s multiple comparisons *post-hoc* test between groups. When assuming normal distribution, parametric tests were used. Multiple paired t-test (one per row) with False Discovery Rate approach were used to compare glucose uptake and lipolysis data between two groups. Two-way ANOVA was used to test for a significant effect of two factors and the interaction, followed by Dunnett’s multiple comparisons *post-hoc* test between groups. *P* < 0.05 was considered statistically significant. EC50 was calculated using GraphPad Prism 8.4.3 software (GraphPad Software). GraphPad Prism 8.4.3 software was used for statistical analysis.

## 3 Results

### 3.1 Doxorubicin, nutlin-3a, and etoposide induce senescence in human adipocytes

**Figure 1:**
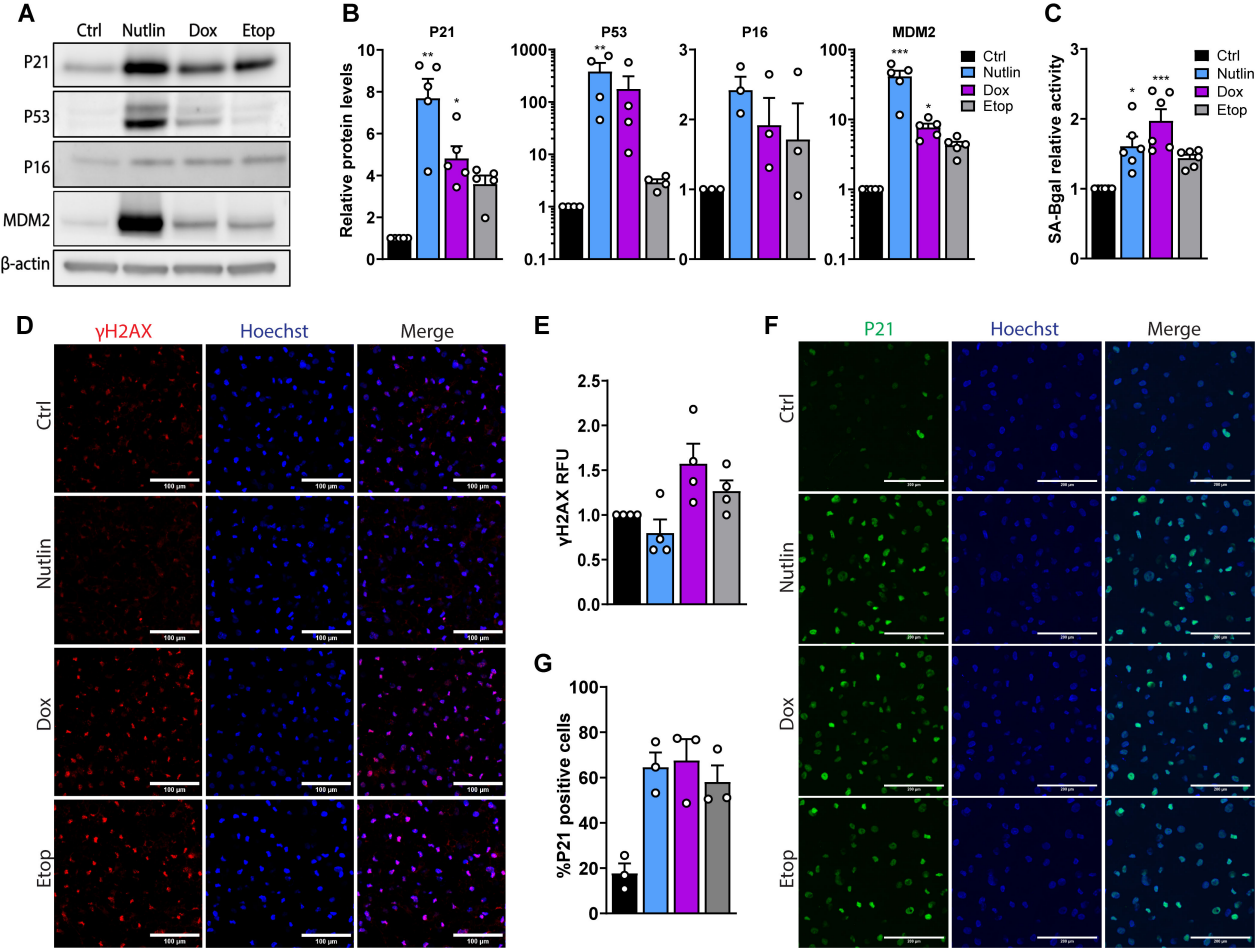
Senescence markers are induced in human adipocytes by treatment with nutlin-3a, doxorubicin, and etoposide. Differentiated human adipocytes were treated with nutlin-3a (Nutlin; 5 µM), doxorubicin (Dox; 0.3 µM) or etoposide (Etop; 50 µM) for 7 days. **(A)** Western blot analysis of senescence markers and β-actin as a loading control, and **(B)** corresponding quantified levels (n = 3-5/group). **(C)** SA-βgal activity (n = 6/group). **(D)** Representative immunofluorescence images of ɣH2AX (red) and nuclei (Hoechst, blue). Scale bars represent 100 µm. **(E)** Quantified ɣH2AX fluorescence intensity (n = 4/group). Values are normalized to quantified nuclei (Hoechst) and expressed as relative fluorescence units (RFU). **(F)** Representative immunofluorescence images of p21 (green) and nuclei (Hoechst, blue). Scale bars represent 200 µm. **(G)** Quantified p21 fluorescence intensity expressed as the percentage of cells positive for p21 (n = 3/group). Values are normalized to quantified nuclei (Hoechst). Data are shown as mean ± SEM. Data were analyzed by Friedman test followed by Dunn’s multiple comparisons test. *P < 0.05, **P < 0.01, ***P < 0.001 *vs.* control. The statistical analysis of ɣH2AX fluorescence intensity **(E)** showed a significant difference between the groups, *P* < 0.05 (Friedman test; but the Dunn’s multiple comparisons test showed no significant differences between individual groups).

To induce cellular senescence, we treated human adipocytes with compounds that have previously been reported to induce senescence in other cell types (21). Human preadipocytes were differentiated for 10 days prior to treatment with a p53 activator (5 µM nutlin-3a, Nutlin) and two DNA-damaging agents (0.3 µM doxorubicin, Dox; 50 µM etoposide, Etop) for 7 days. Nutlin and Dox induced expression of senescence markers p21, p53 and MDM2 (**Figures 1A, B**). Nutlin induced a 7-fold increase of p21 protein levels, and a 40-fold and 380-fold increase of MDM2 and p53 protein levels, respectively. Although not statistically significant, the expression of senescence markers after Etop treatment exhibited a pattern similar to those observed with Nutlin and Dox treatment. In addition, treatment with Dox, Etop, and Nutlin increased the activity of Senescence-associated β-galactosidase (SA-βgal) in the adipocytes by 1.4- to 2-fold (**Figure 1C**). Furthermore, all three compounds at the given concentrations (especially Nutlin and Dox) caused morphological changes in the human adipocytes, which became more elongated and fibroblast-like (**Supplementary Figure 1B**).

DNA damage is a trigger of senescence which activates a DNA damage response (5). We investigated the expression of Ser139 phosphorylated H2AX (ɣH2AX) – a marker of double-stranded DNA breaks (22). Immunostaining revealed that ɣH2AX expression was 1.6- and 1.3-fold higher in cells treated with the DNA damaging agent Dox and Etop, respectively (**Figures 1D, E**), in addition to confirming the elevated expression of p21 in treated cells (**Figures 1F, G**). The number of p21-positive adipocytes increased from 20% in control cells to 60-80% in adipocytes treated with Dox, Etop and Nutlin (**Figure 1G**). To validate that the induced senescence phenotype was stable compounds were washed out at day 7 of treatment. Indeed, the number of p21-positive cells was still around 70% four days after compounds had been washed out (**Supplementary Figure 1C**). Taken together, these findings demonstrate that induction of senescence in human adipocytes can be achieved following treatment with Dox, Etop, and Nutlin.

### 3.2 Senescent human adipocytes develop a SASP

**Figure 2:**
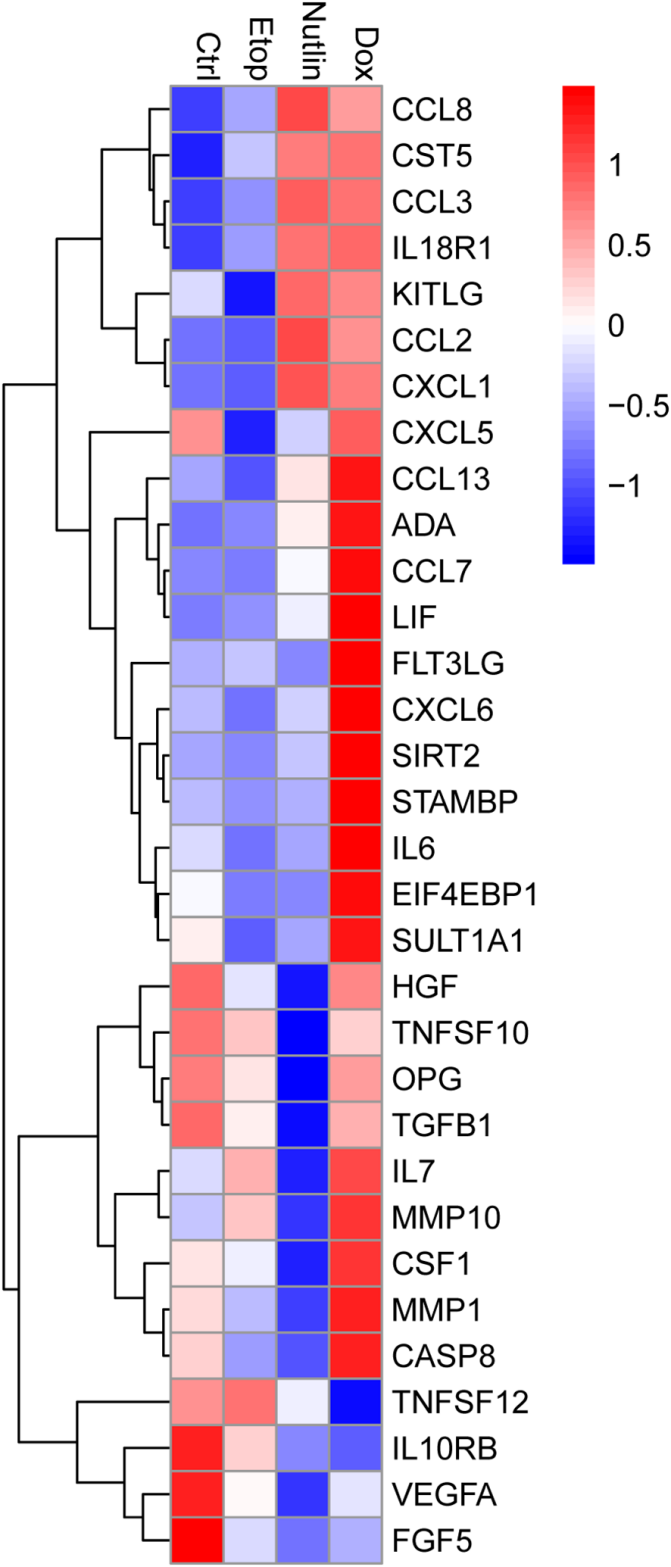
Senescent adipocytes secrete SASP factors. Heat map displaying secreted inflammatory factors in cell media collected from adipocytes treated with nutlin-3a (Nutlin; 5 µM), doxorubicin (Dox; 0.3 µM) or etoposide (Etop; 50 µM) for 7 days (n = 6/group). Displayed analytes showed significant variance between groups (Friedman test; *P* < 0.05; n = 6). Color intensity represents z-score.

To study the SASP of senescent human adipocytes, the composition of inflammatory proteins was assessed in cell media from senescent adipocytes using a targeted inflammatory proteome platform. Proteins that displayed a statistically significant difference (Friedman test) between treatment groups are shown in **Figure 2** and are clustered based on similar responses to treatment. Although the strongest response was observed in Dox-treated adipocytes, increased secretion of inflammatory proteins could be detected in cell media from adipocytes treated with all three compounds (**Figure 2**). The senescence-inducing compounds increased the secretion of several known SASP factors, including IL6, CCL2, CCL3, CCL7, CCL8, CCL13, CXCL1, CXCL5, KITLG, CSF1, MMP1, and MMP10 (18, 23–26). Other factors that were increased, including CASP8, ADA and FLT3LG, have not, to the best of our knowledge, been appointed as SASP previously. Furthermore, secretion of other proteins, including FGF5, TGFB1, OPG and the angiogenic factor VEGFA were decreased in senescent adipocytes.

### 3.3 Stimulated lipolysis is not altered in senescent human adipocytes

**Figure 3:**
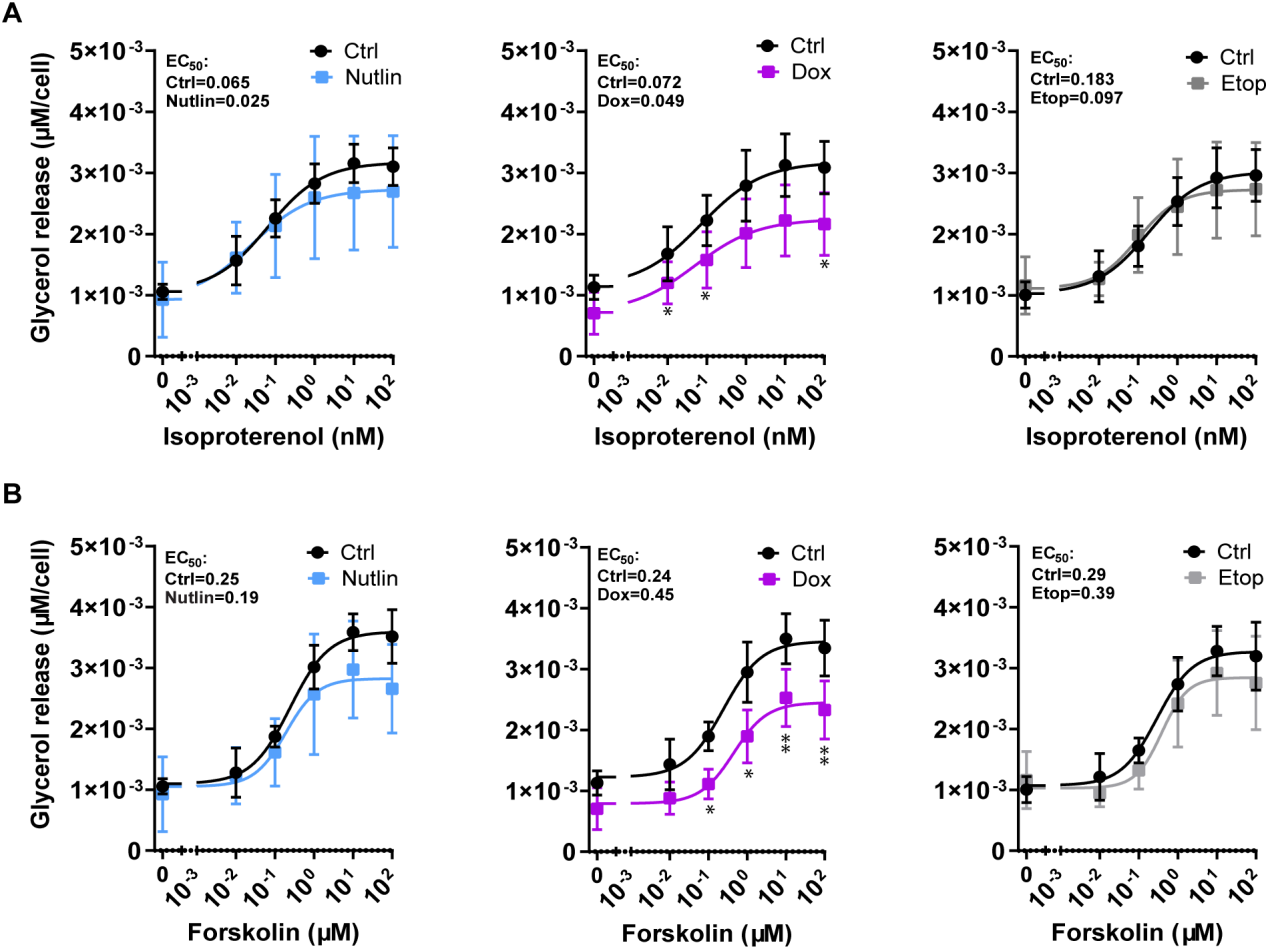
Stimulated Lipolysis remains unchanged in senescent adipocytes. Differentiated human adipocytes were treated with nutlin-3a (Nutlin; 5 µM), Doxorubicin (Dox; 0.3 µM) or etoposide (Etop; 50 µM) for 7 days. **(A)** Basal (n = 2/group) and isoproterenol-stimulated lipolysis (n = 3/group). **(B)** Basal (n = 2/group) and forskolin-stimulated lipolysis (n = 3/group). Data are shown as mean ± SEM. Data were analyzed by multiple paired t-test with False Discovery Rate approach. Basal values were excluded from statistical analyses.

To determine if cellular senescence has an impact on lipolysis, human adipocytes treated with Dox, Etop, and Nutlin for 7 days were exposed to isoproterenol or forskolin. Isoproterenol stimulates lipolysis by binding and activating β-adrenergic receptors, while forskolin stimulates adenylyl cyclase leading to increased lipolytic activity (27, 28). Lipolysis was determined as the release of glycerol in cell culture media. Isoproterenol stimulation yielded a numerically lower EC50 in senescent adipocytes compared with control cells, whereas this was not consistently observed with forskolin stimulation (**Figures 3A, B**). Maximal stimulated lipolysis, both with forskolin and isoproterenol stimulation, trended to be slightly lower in senescent adipocytes compared to control cells. No significant difference in basal or stimulated lipolysis was found between controls and Nutlin- and Etop-treated cells using isoproterenol (**Figure 3A**) or forskolin (**Figure 3B**). However, Dox-treated adipocytes displayed a reduction in glycerol release with both forskolin and isoproterenol stimulation compared to control cells. The lower lipolysis by Dox-treated adipocytes is most likely explained by a reduction in basal lipolysis. These results suggest that the sensitivity to stimulation of lipolysis and lipolytic capacity of senescent adipocytes remain largely unaffected.

### 3.4 Insulin-stimulated glucose uptake is reduced in senescent human adipocytes

**Figure 4:**
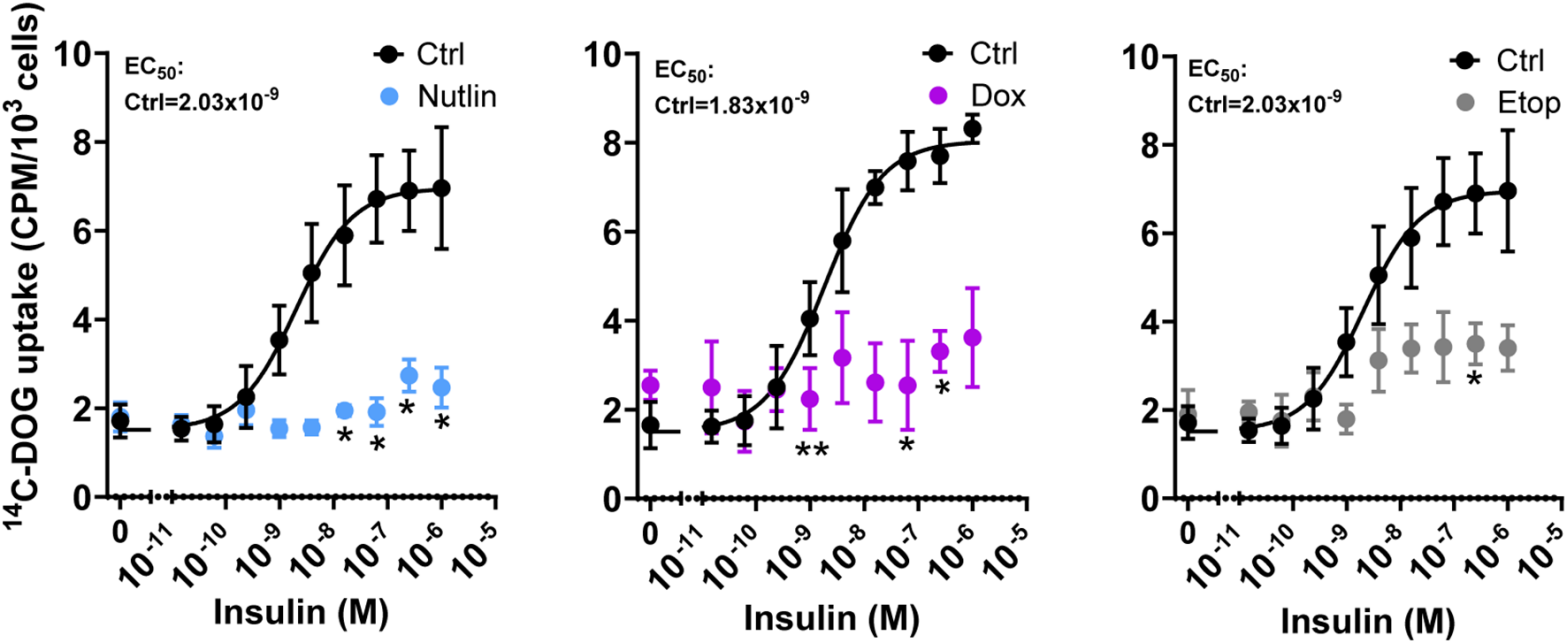
Cell senescence reduces insulin-stimulated glucose uptake in human adipocytes. Differentiated human adipocytes were treated with nutlin-3a (Nutlin; 5 µM), doxorubicin (Dox; 0.3 µM) or etoposide (Etop; 50 µM) for 7 days. Basal and insulin-stimulated glucose uptake (nutlin-3a and etoposide n = 4; doxorubicin n = 3). Data are shown as mean ± SEM. Data were analyzed by multiple paired t-test with False Discovery Rate approach. *P < 0.05, **P < 0.01 *vs.* control cells.

Next, we investigated if cellular senescence impacts basal and insulin-responsive glucose uptake in adipocytes by measuring uptake of a ^14^C-labeled glucose analogue, deoxy-D-glucose. To stimulate glucose uptake, we exposed Dox-, Etop-, and Nutlin-treated adipocytes to insulin for 45 min. As expected, insulin stimulation resulted in a dose-dependent increase in glucose uptake in control cells with an EC_50_ of ~2 nM (**Figure 4**). Whereas the basal glucose uptake of control and senescent cells were similar, insulin-stimulated glucose uptake was markedly decreased in senescent adipocytes. Treatment with Dox-, Etop-, and Nutlin caused a reduction of the maximal insulin-stimulated glucose uptake by 66-82% compared to control treatment. Thus, these results indicate that insulin-stimulated glucose uptake in senescent human adipocytes is impeded.

### 3.5 Insulin receptor signaling remains largely intact in senescent adipocytes

**Figure 5:**
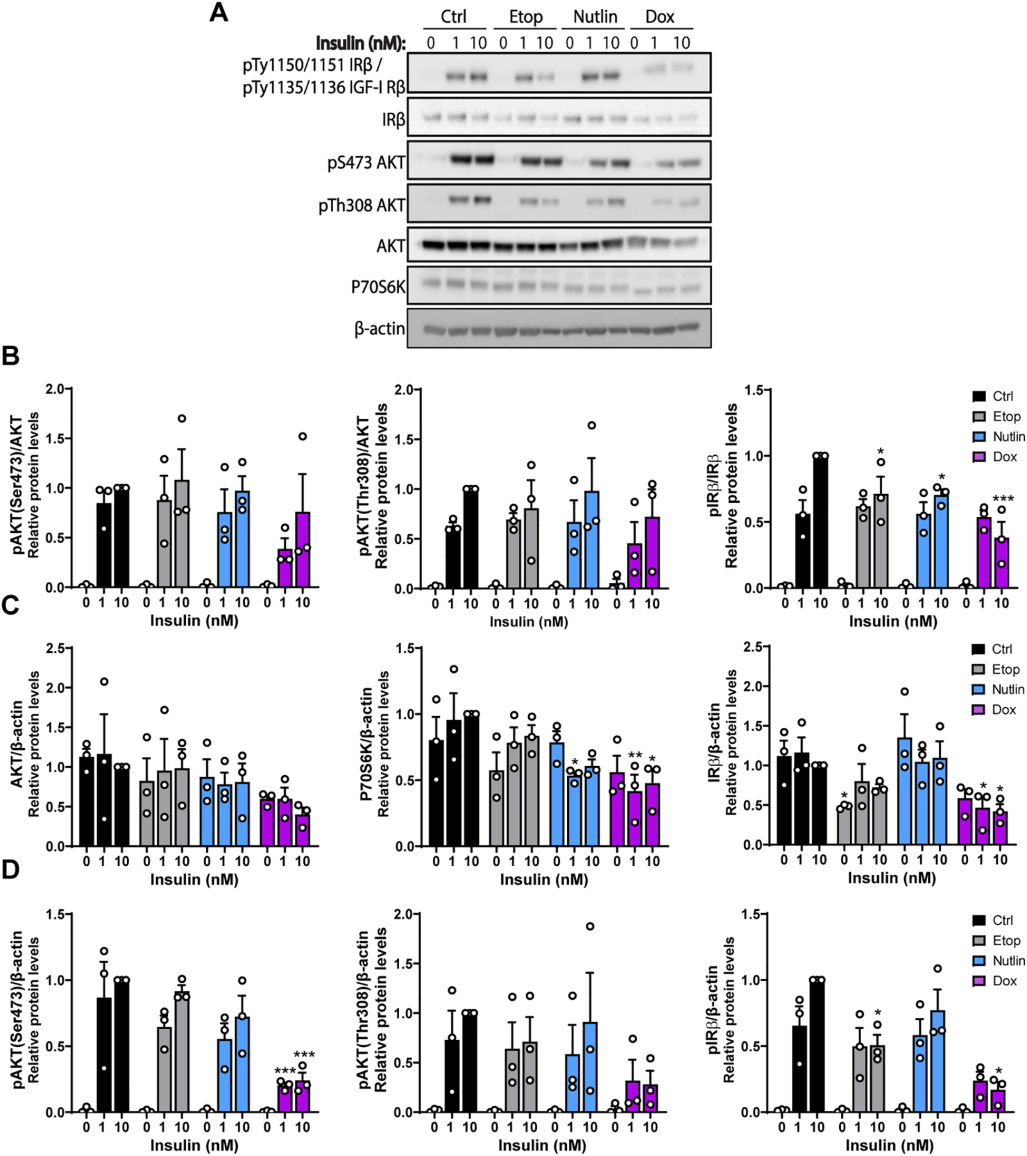
Insulin signaling remains largely intact in senescent human adipocytes. Differentiated human adipocytes were treated with nutlin-3a (Nutlin; 5 µM), doxorubicin (Dox; 0.3 µM) or etoposide (Etop; 50 µM) for 7 days. **(A)** Western blot analysis of insulin signaling proteins and β-actin as a loading control. **(B)** Quantified phosphorylated protein levels (pAKT(Ser473), pAKT(Thr308) and pIRβ). Protein levels are normalized to respective total protein. **(C)** Quantified protein levels (AKT, P70S6K and IRβ) normalized to β-actin. **(D)** Quantified phosphorylated protein levels (pAKT(Ser473), pAKT(Thr308) and pIRβ). Protein levels are normalized to β-actin. Data are shown as mean ± SEM, (n = 3/group). Data were analyzed by two-way ANOVA followed by Dunnett’s multiple comparisons test. *P < 0.05, **P < 0.01, ***P < 0.001 *vs.* control cells stimulated with 10 nM insulin.

Reduced insulin-stimulated glucose uptake could be a consequence of reduced insulin receptor signaling. Key insulin signaling proteins and their phosphorylation were measured in human adipocytes treated with the senescence-inducing compounds, followed by insulin stimulation for 10 min (**Figure 5A**). Expression of AKT, insulin receptor β (IRβ) and P70S6K could be detected in samples from both control and senescent cells, as well as phosphorylated levels of IRβ (Tyr1150/1151) and AKT (Ser473 and Thr308) (**Figure 5A**). The ratio of phosphorylated levels of AKT (Ser473 and Thr308) and IRβ (Tyr1150/1151) over total protein were similar between control and senescent cells at 1nM insulin stimulation, indicating preserved receptor sensitivity to insulin. However, incubating Dox-treated cells with 10 nM of insulin yielded a reduced pIRβ response (**Figure 5B**). Dox-treatment reduced the total protein levels of both IRβ and P70S6K (**Figure 5C**). Investigating the phosphorylated proteins with β-actin ratio showed that dox-treated adipocytes had a 76% reduction in phosphorylation of AKT(Ser473) and 83% in phosphorylation of IRβ (Tyr1150/1151) at 10 nM of insulin (**Figure 5D**). This finding indicates that the phosphorylation ratio in Dox-treated adipocytes visualized in **Figure 5B**, is overestimating the total amount of phosphorylated signaling proteins in dox-treated cells. Together, these results indicate that the insulin action capacity in senescent adipocytes induced by dox is reduced, while the receptor signaling sensitivity to insulin is generally preserved.

### 3.6 Cellular senescence in human adipocytes reduces expression of GLUT4 and adipocyte marker genes

**Figure 6:**
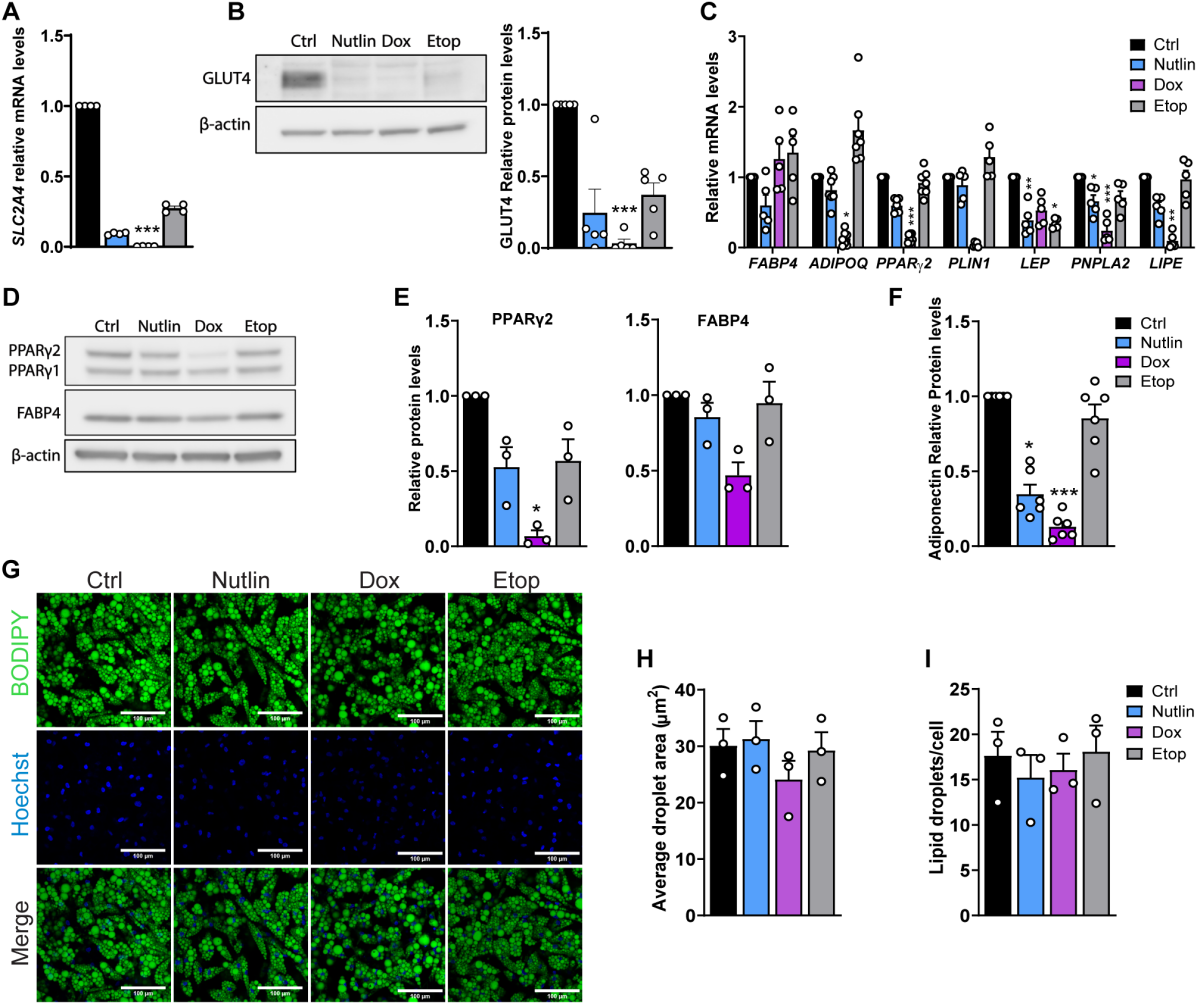
Senescent adipocytes have reduced expression of adipocyte markers. Differentiated human adipocytes were treated with nutlin-3a (Nutlin; 5 µM), doxorubicin (Dox; 0.3 µM) or etoposide (Etop; 50 µM) for 7 days. **(A)** mRNA levels of *SLC2A4* (encoding GLUT4) normalized to *TBP* (n = 4/group). **(B)** Western blot analysis of GLUT4 and β-actin as a loading control, and quantified protein levels (n = 5/group). **(C)** mRNA levels of adipocyte markers *FABP4, ADIPOQ*, *PPARɣ2*, *PLIN1*, *LEP*, *PNPLA2*, and *LIPE* normalized to *TBP* (*FABP4*, *PLIN1*, *LEP*, *PNPLA2*, and *LIPE* n = 5/group; *ADIPOQ* and *PPARɣ2* n = 7/group). **(D)** Western blot analysis of PPARɣ, FABP4 and β-actin as a loading control. **(E)** Quantified protein levels of PPARɣ and FABP4 normalized to β-actin (n = 3/group). **(F)** Adiponectin levels in cell culture media (n = 6/group). **(G)** Representative immunofluorescence images of lipid droplets (BODIPY; green) and nuclei (Hoechst, blue). Scale bars represent 100 µm. **(H)** average lipid droplet area (µm^2^; n=3/group). **(I)** Quantified number of lipid droplets per cell (n = 3/group). Data are shown as mean ± SEM. Data were analyzed by Friedman test followed by Dunn’s multiple comparisons test. *P < 0.05, **P < 0.01, ***P < 0.001 *vs.* control cells.

GLUT4 is the glucose transporter mediating insulin-responsive glucose uptake. We therefore investigated GLUT4 expression and found that treatment with Dox, Nutlin and Etop reduced the expression of GLUT4 mRNA (encoded by *SLC2A4*) by 70 to almost 100%, and GLUT4 protein level by 60 to almost 100% (**Figures 6A, B**). The marked decrease in GLUT4 expression likely contributes to the reduced insulin-stimulated glucose uptake in senescent adipocytes.

Given the reduced GLUT4 mRNA and protein expression, and the slightly lower total expression of insulin-responsive proteins, especially IRβ, we hypothesized that the adipocytes could be partly dedifferentiated as they become senescent. To investigate this further, the expression of adipocyte markers was measured. At the mRNA level, Dox treatment reduced the expression of adiponectin (*ADIPOQ)* and perilipin1 *(PLIN1)* (**Figure 6C**). Both Dox and Nutlin reduced the mRNA levels of *PPARɣ2 - a* key regulator of adipogenesis, while Nutlin and Etop decreased levels of leptin (*LEP*) (**Figure 6C**). Expression of ATGL (*PNPLA2)* and HSL (*LIPE)*, two key lipolytic enzymes, were reduced by both Nutlin and Dox-treatment (**Figure 6C**). At the protein level, Dox but not Nutlin and Etop treatment lowered expression of PPARɣ2 (**Figures 6D, E**). Furthermore, secreted levels of adiponectin were found to be reduced by 65-87% in senescent human adipocytes treated with Nutlin and Dox compared to controls (**Figure 6F**). Last, we used imaging to evaluate whether senescence impacted adipocyte lipid content and lipid droplet morphology as measured by lipid droplet number and size (**Figures 6G–I**). No difference in lipid droplet number or size was observed between senescent adipocytes and control cells. These findings suggest that some, but not all, characteristics of the adipocyte phenotype are altered by senescence.

## 4 Discussion

WAT dysfunction is a central mechanism in obesity-related metabolic diseases. We recently showed both *in vivo* and *in vitro* that mature human adipocytes can undergo senescence in response to hyperinsulinemia (18). Furthermore, senescence markers are elevated in mature adipocytes isolated from obese individuals, particularly in individuals with obesity and T2D (17). The contribution of adipocyte senescence to obesity-related metabolic diseases like T2D is, however, not well understood. In the current study, we explored the effect of cellular senescence on adipocyte metabolic function and its possible link to metabolic dysfunction. By using different models to induce senescence in human adipocytes, we showed that senescent human adipocytes display markedly reduced insulin-stimulated glucose uptake but retain normal lipolytic capacity and insulin receptor signaling. Adipocyte senescence further led to reduced expression of adipogenic differentiation markers, including GLUT4.

Senescence can be induced by a variety of different stimuli. Here we used three compounds to induce senescence in human adipocytes, with different senescence-inducing mechanisms, i.e., nutlin-3a, a p53-activator, and doxorubicin and etoposide, two DNA-damaging agents (29–31). The selection of compounds was based on existing literature confirming their senescence-inducing properties in other cell types, including cell lines and primary cells (21, 32, 33). All treatments induced an increase in several senescence markers (e.g., p21, p53, SASP, and SA-βgal activity) after 7 days of treatment. Treatment with doxorubicin and Nutlin produced a stronger response in several of the measured parameters than etoposide. Etoposide treatment showed a similar pattern, albeit less prominent, and caused metabolic changes like those observed with Dox and Nutlin.

The senescent phenotype is complex and dynamic, with phenotypic variations depending on the type of stress that initially triggered the cell to enter senescence. All our readouts and functional studies were conducted after 7 days of treatment with the senescence-inducing drugs. It is possible that investigations at earlier or later time points might lead to differences in results compared to ours. The impact on glucose uptake and GLUT4 levels was observed after treatment with all three compounds, increasing our confidence that the observed effects are a consequence of senescence induction, rather than compound-specific effects. In addition, washing out the compounds did not impact the expression of p21. Even though all three compounds induced a SASP, differences in composition and number of detected inflammatory molecules in cell media differed between the three inducers. Indeed, the heterogeneity of SASP has previously been demonstrated to not only depend on cell type, but also on the senescence stressor (23, 33, 34). Several known SASP factors were secreted by the senescent adipocytes. Other factors that were increased, including caspase 8 (CASP8), ADA, CST5, STAMBP and FLT3LG, have, to the best of our knowledge, not been appointed as SASP previously. One of these factors, CASP8, was recently found to be increased in perigonadal adipose tissue from HFD mice, and in omental adipocytes from individuals with type 2 diabetes (35). In the same study, adipocyte specific CASP8 knockout mice fed a high-fat diet had improved glucose tolerance, suggesting a link between caspase 8 in adipocytes and dysregulated glucose metabolism. The increased secretion of adenosine deaminase (ADA) is noteworthy, as ADA promotes lipolysis. We found lipolysis to be largely unaffected by senescence, especially in Nutlin and Etop treated cells, suggesting that the secretion of ADA as SASP does not directly increase lipolysis in senescent adipocytes. It is however possible that secretion of ADA by senescent adipocytes promotes lipolysis of non-senescent surrounding adipocytes, but this needs to be further investigated. Another factor, SIRT2, previously identified to be upregulated in senescence induced by Dox, was also found to be secreted by Dox-induced senescent adipocytes (36). The divergent SASP response, while similar response to doxorubicin, nutlin-3a and etoposide on insulin-stimulated glucose uptake and stimulated lipolysis indicate that SASP had minor influence on these metabolic effects.

A hallmark of T2D is the impaired response to insulin measured as reduced insulin-stimulated glucose uptake. In the current study, we found that cellular senescence markedly reduces insulin-stimulated, but not basal, glucose uptake in human adipocytes. The markedly reduced insulin-stimulated glucose uptake prohibited calculation of EC50, and therefore any change in insulin sensitivity could not be determined. However, the markedly reduced response to insulin suggests that the senescent adipocytes have become insulin resistant. The senescent adipocytes had distinctly lower expression of GLUT4. Reduced expression of GLUT4 was also observed in adipose tissue from humans with T2D and obesity, and in adipocytes isolated from individuals with obesity and T2D associated with increased expression of senescence markers (17, 37). Our results are in line with a study in which Nutlin- and Dox-treated differentiated murine 3T3-L1 cells and differentiated human visceral preadipocytes showed reduced uptake of glucose in response to insulin (38). In addition, Ge et al., recently showed that differentiated preadipocytes isolated from inguinal adipose tissue from high-fat diet treated, and naturally aged rats, displayed reduced glucose uptake capacity (39). In both studies, the murine cells exhibited decreased GLUT4 levels at plasma membrane (38, 39). Together with previous findings, our results suggest that cellular senescence in adipocytes may contribute to the impaired glucose tolerance associated with obesity and T2D.

We hypothesized that the observed reduction in insulin-stimulated glucose uptake could be a consequence of impaired insulin receptor signaling in senescent adipocytes. Although we did find that particularly the Dox-treated cells had reduced levels of insulin-responsive proteins (IRβ, P70S6K), interestingly, senescent adipocytes retain their sensitivity to insulin as measured by phosphorylation of AKT and IRβ. It is unlikely that the reduced levels of insulin signaling proteins explains the robust decrease in insulin-stimulated glucose uptake since the total amount of phosphorylated proteins was mainly reduced in Dox treated cells, but all senescence treatments produced a similar reduction in insulin-mediated glucose uptake. However, it cannot be ruled out that the dox-induced reductions in insulin-responsive proteins contribute to reduced glucose uptake in dox-induced senescence. These results contrasts with our recent findings showing that cellular senescence induced by Dox, Nutlin and Etop in human hepatocyte cell lines (HepG2 and IHH cells) rather enhances insulin receptor signaling and emphasize that the senescent phenotype may vary depending on cell type (32).

We observed no major difference in basal or stimulated glycerol release when inducing lipolysis using the β-adrenergic antagonist isoproterenol or by using forskolin in cells exposed to nutlin-3a and etoposide. Our results indicate that senescent adipocytes remain responsive to β-adrenergic stimuli. However, Dox-treated adipocytes displayed reduced lipolysis at all doses of isoproterenol or forskolin, while the EC50 was similar, suggesting reduced basal lipolysis. *PNPLA2* and *LIPE* mRNA levels – encoding key lipolytic enzymes ATGL and HSL - were significantly decreased by Dox treatment. Surprisingly, Nutlin also significantly reduced *PNPLA2* mRNA levels even though lipolysis was unchanged. This may reflect differences between protein and mRNA levels, or changes in activation of these proteins. These results are in contrast with previous findings in which Nutlin- and Dox-treated differentiated murine 3T3-L1 cells and differentiated human visceral preadipocytes displayed an increase in basal lipolysis (38). It is possible that the effect of senescence on basal lipolysis differs between differentiated 3T3-L1 cells and subcutaneous human adipocytes, and between senescent adipocytes from different adipose depots. The observed differences in results might also reflect the dynamic changes in the senescent phenotype over time as the lipolytic measurements were performed at different timepoints after induction of senescence. Our results indicate that senescence does not impact all aspects of adipocyte metabolism and function but selectively affects insulin-mediated glucose uptake and not stimulated lipolysis. It could be speculated that the reduced insulin-stimulated glucose uptake, would contribute to reduced esterification of fatty acids to triglycerides in adipocytes post meals resulting in increased flux of fatty acids to other tissues thereby enhancing insulin resistance and ectopic fat accumulation.

Expression of adipocyte markers PPARɣ, GLUT4, *LEP*, *PNPLA2*, *LIPE*, and adiponectin were all reduced in senescent adipocytes. However, there was no difference in the amount and size of lipid droplets across all treatments, suggesting that the senescence-inducing compounds cause a shift towards a less differentiated state. Reduced expression of PPARɣ and adiponectin in senescent human adipocytes have previously been reported (17), but selective sparing of lipolysis and markedly reduced insulin-stimulated glucose uptake associated with reduced GLUT4 levels have, to the best of our knowledge, not been shown before.

Our observation that senescent adipocytes display diminished glucose uptake aligns with findings from studies in which senescent cell burden has been reduced by senolytics or using transgenic mice. Elimination of senescent cells by treatment with senolytics dasatinib and quercitin improved glucose homeostasis in diet-induced obese mice, db/db mice, and aged mice (9, 40, 41). Similarly, enhanced glucose tolerance was achieved by depletion of senescent cells using transgenic mouse models targeting p16- or p21-positive cells (9, 42).

A limitation of the study is the lack of *in vivo* experiments. All results presented here have been generated using primary human differentiated preadipocytes, and do not reflect the complexity of adipose tissue *in vivo* and the cross talk between different cell types. It should also be noted that the preadipocytes used in this study are from non-obese individuals and may not fully capture the senescent phenotype of senescent adipocytes in obesity and T2D.

In summary, our results demonstrate that cellular senescence selectively impairs adipocyte function by reducing insulin-stimulated glucose uptake. Our results further indicate that senescent adipocytes remain responsive to insulin and retain their lipolytic capacity. Adipocyte senescence may be a contributing factor in the development of adipose tissue dysfunction observed in metabolic disease.

## Data Availability

The original contributions presented in the study are included in the article/**Supplementary Material**. Further inquiries can be directed to the corresponding author.
