# Supplementary material for: Insulin-stimulated glucose uptake is impaired in senescent human adipocytes
Source: Front Endocrinol (Lausanne). 2026 Mar 17;17:1795654. doi: 10.3389/fendo.2026.1795654 (PMC13035520; doi:10.3389/fendo.2026.1795654)
Supplement: Supplementary file 1 [file DataSheet1.docx]

Supplementary data

**Supplementary Figure 1**


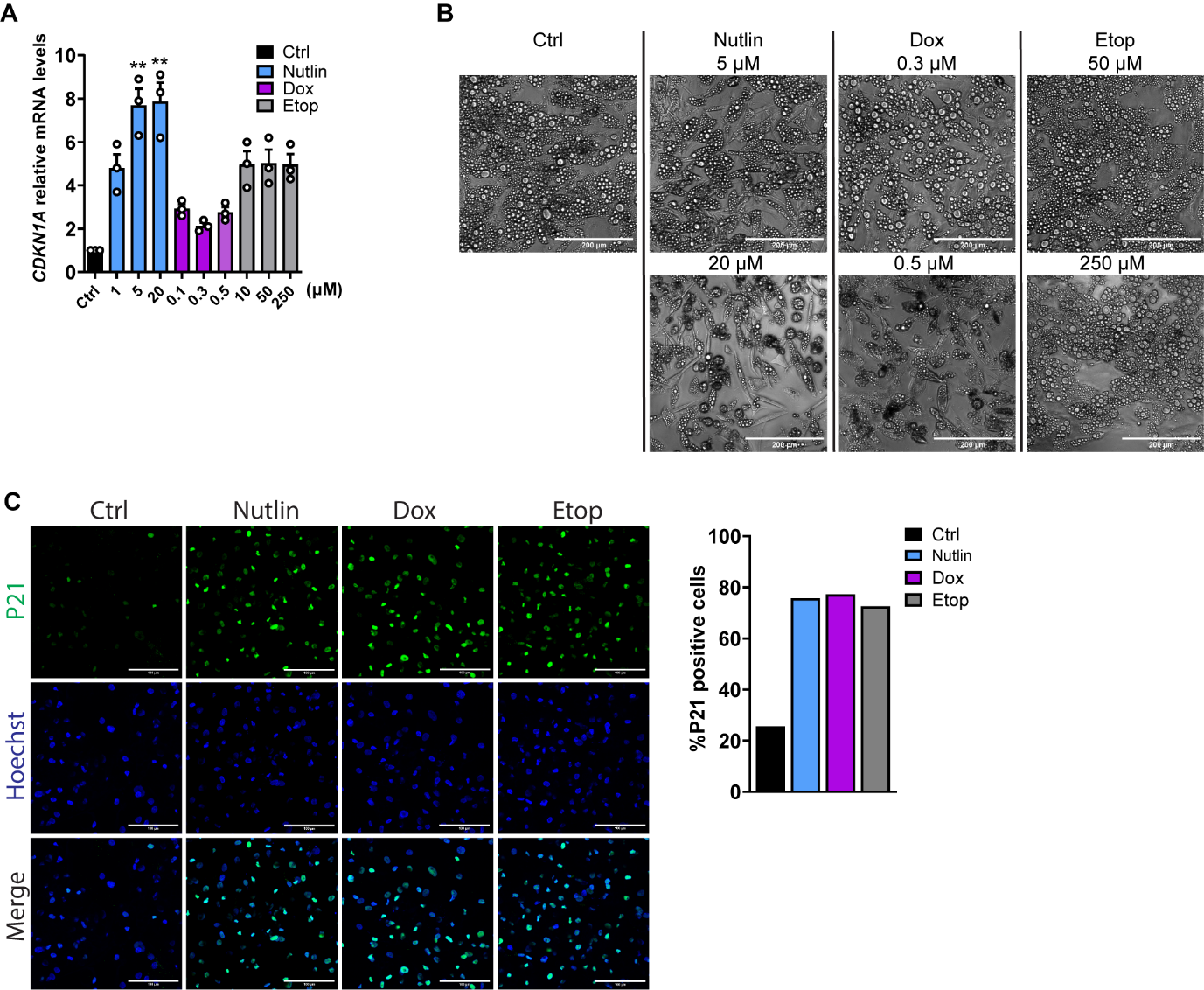


**Supplementary Figure 1. Senescent adipocyte morphology and p21 levels after washout of compounds.** Differentiated human adipocytes were treated with nutlin-3a (Nutlin; 5 µM), doxorubicin (Dox; 0.3 µM) or etoposide (Etop; 50 µM) for 7 days. (A) mRNA levels of *CDKN1A* (encoding p21) normalized to *TBP* (n = 3/group). (B) Representative phase-contrast images. Scale bars represent 200 µm. (C) Immunofluorescence images of p21 (green) and nuclei (Hoechst, blue) 4 days after compounds have been washed out of the cell cultures. Scale bars represent 200 µm. Quantified p21 fluorescence intensity expressed as the percentage of cells positive for p21 (n = 1/group). Values are normalized to quantified nuclei (Hoechst). Data are shown as mean ± SEM. Data were analyzed by Friedman test followed by Dunn’s multiple comparisons test. **P* < 0.05, ***P* < 0.01, *vs.* control cells.

**Supplementary Table 1. Anthropometric characteristics of subjects**

| **Characteristic** |  |
| --- | --- |
| No. | 10 |
| Sex  Female  Male | 10  0 |
| Age (years) | 50.8 ± 5.7 |
| Weight (kg) | 74.9 ± 7.8 |
| Height (m) | 1.65 ± 0.1 |
| BMI (kg/m^2^) | 26.8 ± 1.8 |

*Data are mean ± SD.*

**Supplementary Table 2. Primer sequences and probes**

| **Gene** | **Species** | **Forward** | **Reverse** | **Product length (base pairs)** |
| --- | --- | --- | --- | --- |
| *TBP* | Human | TTTGCTGCGGTAATCATGAGG | GCTGGAAAACCCAACTTCTGT | 155 |
| *SLC2A4* | Human | TCATCATTGGCATGGGTTTCC | CAGCTGAGATCTGGTCAAACG | 150 |
| *PPARɣ* | Human | GACCCAGAAAGCGATTCCTTC | TCCATTACGGAGAGATCCACG | 131 |
| *FABP4* | Human | CATGTGCAGAAATGGGATGGA | CGAACTTCAGTCCAGGTCAAC | 151 |
| *ADIPOQ* | Human | TGGTGAGAAGGGTGAGAAAGG | CTCCAATCCCACACTGAATGC | 175 |

| **Gene** | **Species** | **Vendor** | **Cat. no** |
| --- | --- | --- | --- |
| *LEP* | Human | Thermo Fisher Scientific | Hs00174877_m1 |
| *PLIN1* | Human | Thermo Fisher Scientific | Hs00160173_m1 |
| *LIPE* | Human | Thermo Fisher Scientific | Hs00943410_m1 |
| *PNPLA2* | Human | Thermo Fisher Scientific | Hs00982042_m1 |

**Supplementary Table 3. Antibodies for Western blot and immunocytochemistry**

| **Antibody for Western blot** | **Supplier** | **Cat. no** | **Dilution** |
| --- | --- | --- | --- |
| AKT | Cell signaling technology | 4691 | 1:1000 |
| β-actin | Sigma-Aldrich | A5441 | 1:5000 |
| FABP4 | Cell signaling technology | 3544 | 1:1000 |
| GLUT4 | Cell signaling technology | 2213S | 1:1000 |
| Insulin receptor β (IRβ) | Cell signaling technology | 3025S | 1:1000 |
| MDM2 | Cell signaling technology | 86934 | 1:1000 |
| p21 Waf1/Cip1 | Cell signaling technology | 2947 | 1:1000 |
| p53 | Cell signaling technology | 2527 | 1:1000 |
| p70S6K | Cell signaling technology | 9202 | 1:1000 |
| pAKT (Ser473) | Cell signaling technology | 4060 | 1:2000 |
| pAKT (Thr308) | Cell signaling technology | 9275S | 1:1000 |
| pIGF/IRβ | Cell signaling technology | 3024S | 1:1000 |
| PPARγ | Cell signaling technology | 2435 | 1:1000 |

| **Antibody for immunocytochemistry** | **Supplier** | **Cat. no** | **Secondary antibody** |
| --- | --- | --- | --- |
| ɣH2AX (Ser139) | Sigma-Aldrich | 05-636 | AlexaFluor Plus 647, ThermoFisher, (A32728) |
| p21 | Abcam | ab109520 | AlexaFluor Plus 647, ThermoFisher, (A32733) |

**Supplementary Table 4. Normalized Protein Expression (NPX) of inflammatory factors in cell media**

|  |  | Normalized Protein Expression (NPX) | | | |
| --- | --- | --- | --- | --- | --- |
| Protein | **Uniprot ID** | **Ctrl** | **Nutlin** | **Dox** | **Etop** |
| VEGFA | P15692 | 12,25 | 10,07*** | 10,98* | 11,12 |
| CCL7 | P80098 | 1,47 | 2,58 | 5,08** | 1,40 |
| IL7 | P13232 | 0,18 | 0,02 | 0,38 | 0,29 |
| OPG | O00300 | 9,52 | 6,44*** | 9,28 | 8,69* |
| TGFB1 | P01137 | 3,35 | 1,53*** | 3,04 | 2,73 |
| IL6 | P05231 | 8,69 | 8,38 | 10,49 | 8,05 |
| CCL2 | P13500 | 13,01 | 14,70* | 14,30 | 12,88 |
| CST5 | P28325 | 2,99 | 3,38** | 3,39* | 3,17 |
| CXCL1 | P09341 | 9,87 | 12,35* | 11,97 | 9,65 |
| KITLG | P21583 | 3,27 | 3,67 | 3,61 | 2,87 |
| CCL13 | Q99616 | 11,04 | 12,20 | 14,10* | 10,34 |
| FGF5 | P12034 | 1,49 | -0,11*** | 0,13* | 0,28 |
| MMP1 | P03956 | 8,71 | 7,55 | 9,61 | 8,13 |
| IL10RB | Q08334 | 0,21 | -0,15* | -0,19** | 0,03 |
| IL18R1 | Q13478 | 1,89 | 2,52* | 2,54 | 2,07 |
| CXCL5 | P42830 | 9,54 | 8,46 | 9,89 | 7,24* |
| HGF | P14210 | 7,47 | 5,91** | 7,35 | 6,75 |
| MMP10 | P09238 | 1,54 | 1,02 | 2,44 | 1,91 |
| CCL3 | P10147 | 2,15 | 4,43** | 4,32* | 2,69 |
| FLT3LG | P49771 | 4,17 | 4,04 | 5,23* | 4,24 |
| CXCL6 | P80162 | 5,35 | 5,56 | 7,91 | 4,74 |
| EIF4EBP1 | Q13541 | 2,84 | 2,33 | 3,86 | 2,31 |
| SIRT2 | Q8IXJ6 | 0,74 | 0,84 | 2,20** | 0,59 |
| LIF | P15018 | 1,25 | 2,52 | 5,70** | 1,46 |
| CCL8 | P80075 | 4,10 | 7,14*** | 6,49* | 4,94 |
| CASP8 | Q14790 | 2,69 | 2,05 | 3,20 | 2,29 |
| TNFSF10 | P50591 | 1,63 | 1,24* | 1,54 | 1,54 |
| TNFSF12 | O43508 | 4,45 | 3,87 | 2,85** | 4,60 |
| SULT1A1 | P50225 | -0,21 | -0,35 | 0,09* | -0,44 |
| STAMBP | O95630 | 1,87 | 1,82 | 3,02* | 1,70 |
| ADA | P00813 | 4,51 | 5,73* | 7,39*** | 4,69 |
| CSF1 | P09603 | 10,36 | 10,13 | 10,53 | 10,32 |

**Supplementary Table 4**. **Normalized Protein Expression (NPX) of inflammatory factors in cell media.** Average NPX values of each analyte and treatment group (Ctrl, Nutlin, Dox, and Etop; n = 6/group). Selected analytes displayed a significant variance between groups (Friedman test; *P* < 0.05). **P* < 0.05, ***P* < 0.01, ****P* < 0.001 *vs.* control cells by Friedman test followed by Dunn’s multiple comparisons test.
